# Pain interference mediates the association between epigenetic aging and grip strength in middle to older aged males and females with chronic pain

**DOI:** 10.3389/fnagi.2023.1122364

**Published:** 2023-03-23

**Authors:** Jessica A. Peterson, Joshua A. Crow, Alisa J. Johnson, Lingsong Meng, Asha Rani, Zhiguang Huo, Thomas C. Foster, Roger B. Fillingim, Yenisel Cruz-Almeida

**Affiliations:** ^1^Pain Research and Intervention Center of Excellence, University of Florida, Gainesville, FL, United States; ^2^Department of Community Dentistry and Behavioral Science, University of Florida, Gainesville, FL, United States; ^3^Department of Biostatistics, University of Florida, Gainesville, FL, United States; ^4^Department of Neuroscience, McKnight Brain Institute, University of Florida, Gainesville, FL, United States; ^5^Genetics and Genomics Program, University of Florida, Gainesville, FL, United States

**Keywords:** pain, disability, epigenetic aging, hand grip strength, physical function

## Abstract

**Introduction:**

Chronic pain is one of the leading causes of disability that may accelerate biological aging and reduce physical function. Epigenetic clocks provide an estimate of how the system ages and can predict health outcomes such as physical function. Physical function declines may be attributed to decreases in muscle quality due to disuse that can be measured quickly and noninvasively using grip strength. The purpose of this study was to explore the associations among self-reported pain, grip strength, and epigenetic aging in those with chronic pain.

**Methods:**

Participants (57.91 ± 8.04 years) completed pain questionnaires, a blood draw and hand grip strength task. We used an epigenetic clock previously associated with knee pain (DNAmGrimAge), and used the subsequent difference of predicted epigenetic age from chronological age (DNAmGrimAge-Difference).

**Results:**

Exploratory pathway analyses revealed that pain intensity mediated the association between DNAmGrimAge-difference and handgrip strength in males only (β = −0.1115; CI [−0.2929, −0.0008]) and pain interference mediated the association between DNAmGrimAge-difference and handgrip strength in males β = −0.1401; CI [−0.3400, −0.0222]), and females (β = −0.024; CI [−0.2918, −0.0020]).

**Discussion:**

Chronic knee pain may accelerate epigenetic aging processes that may influence handgrip strength in older age adults. Chronic pain could be a symptom of the aging body thus contributing to declines in musculoskeletal function in later life.

What this paper adds

1. Chronic pain may play a role in accelerated biological aging and strength declines.
2. Pain severity mediates the association between aging and grip strength in males only.
3. Pain interference mediates the relationship between aging and grip strength in males and females.

Applications of study findings

1. Diet and exercise can alter our epigenome and have positive prophylactic effects on muscle morphology to potentially improve muscle strength.
2. Exercise interventions have shown to be successful in attenuating and even reversing the effects of muscle atrophy and strength declines in aging adults, thus improving quality of life and potentially increasing lifespan.

## Introduction

Chronic musculoskeletal pain is among the most common causes of disability in the world (Blyth and Noguchi, 2017). The increased disability associated with chronic musculoskeletal pain may stem from several sources, such as worsened cognition or impaired physical function, both of which have been extensively observed among individuals with chronic pain (Weiner et al., 2006; McGuire, 2013; de Kruijf et al., 2015; Van Der Leeuw et al., 2016; Hicks et al., 2017). While impaired physical function is associated with chronic musculoskeletal pain, declines in physical performance naturally occur as we age. It is thought that chronic pain may indirectly hasten declines in system integrity that lead to losses in physical function (Badley et al., 1994), thus contributing to accelerated biological aging (Sibille et al., 2017). Consequently, pain and aging should be examined collectively when addressing physical functional limitations in older adults.

Chronological age is a poor predictor of functional decline due to the high variability in aging phenotypes (AihieSayer et al., 1999). Using biomarkers to define functional or physiological age-related declines in physical performance may provide a better estimate of how the system ages. As such, recent research efforts have focused on the development of epigenetic “clocks” to estimate biological aging trajectories using the methylation status of specific DNA sites across the genome (Horvath, 2013; Levine et al., 2018; Lu et al., 2019). Various types of epigenetic clocks have improved estimates of health outcomes that change with advancing age, such as physical function (Marioni et al., 2015), obesity (de Toro-Martín et al., 2019), mortality (Grant et al., 2017), and even chronic pain (Cruz-Almeida et al., 2019, 2022). DNAmGrimAge, for example has been found to be highly associated with a variety of health related metrics compared to other epigenetic clocks (Lu et al., 2019), including physical performance in those with chronic pain (Peterson et al., 2022a). This particular clock takes into consideration behavior and uses a linear combination of chronological age, sex, and DNAm-based surrogate biomarkers for several plasma proteins and smoking pack-years (Lu et al., 2019).

As we age, physical performance declines, in part, due to decreases in muscle quantity and quality (Barbat-Artigas et al., 2014; Fragala et al., 2015). One increasingly common method of measuring physical performance, specifically skeletal muscle strength, is handgrip dynamometry (den Ouden et al., 2011). Isometric handgrip strength (HGS) has been overwhelmingly validated as an analog of global strength in most populations (Jakobsen et al., 2010; Stevens et al., 2012; Haider et al., 2016; Cronin et al., 2017). As a brief, low-risk, and easily administered method requiring minimal equipment, HGS has emerged as the prevalent strength measure of older adults in both clinical and research settings to evaluate age-related loss of physical capacity. Furthermore, evidence favors DNAmGrimAge estimates of biological aging as the most predictive “clock” for physical performance, whereby reductions in physical function are associated with an older epigenome (Lu et al., 2019; McCrory et al., 2020; Peterson et al., 2022a). However, the limited available data examining associations of epigenetic age specifically with HGS indicate no association (McCrory et al., 2020), or an association in females only (Peterson et al., 2021).

To better understand the relationship between biological aging and musculoskeletal performance, it is important to examine factors that may lead to impaired physical performance such as chronic pain, especially in terms of pain intensity and pain-related disability. While some data on epigenetic aging and physical function exist (McCrory et al., 2020; Föhr et al., 2021), limited research has examined the relationship of handgrip strength and DNAmGrimAge, and no data exist on this relationship in those with chronic pain. Furthermore, strong evidence suggests that the burden of the pain experience does not fall equally across the biological sexes (Mogil, 2012; Bartley and Fillingim, 2013), that males and females differ in strength (Mathiowetz et al., 1985; Clarke, 1986; Bishop et al., 1987; Jones et al., 2021), and that these patterns appear to be maintained across chronological age. As such, this study sought to explore the associations among self-reported pain, grip strength, and epigenetic aging in those with chronic pain by using regression based moderated-mediation models to examine the influence of self-reported pain on hand-grip strength mediated by epigenetic aging and moderated by biological sex. Based on the limited research in this area, we expected to identify associations between epigenetic aging and strength in community dwelling adults with chronic pain. Specifically, we expected to observe poorer strength performance associated with advanced epigenetic aging and increased chronic pain.

## Materials and methods

### Participants

Participants aged between 45 and 85 years (57.91 ± 8.04) and reported experiences of knee pain lasting at least 3 months, were recruited from two collaborating universities; University of Florida and the University of Alabama, Birmingham. The participants used in this cross sectional study were recruited as part of a parent study that was originally designed to examine race group differences in physical symptoms, psychosocial functioning, and pain-related central nervous system structure and functioning in those with knee pain and an ancillary study that examined epigenetic biomarkers in the same study sample. The institutional review boards had approved the study at both study locations. Study procedures and sample characteristics using different variables collected throughout the project have been reported previously (Sibille et al., 2017; Booker et al., 2019; Cruz-Almeida et al., 2019, 2022; Johnson et al., 2022). Individuals self-identified as non-Hispanic and “African American/Black” or “White/Caucasian/European” and were English speaking. Exclusion criteria for the study was as follows; (1) significant surgery to the index (i.e., most painful) knee (e.g., total knee replacement surgery); (2) cardiovascular disease or history of acute myocardial infarction; (3) uncontrolled hypertension (*blood pressure* > 150/95 mmHg); (4) systemic rheumatic diseases (e.g., rheumatoid arthritis, systemic lupus erythematosus, and fibromyalgia); (5) neuropathy; (6) chronic opioid use; (7) serious psychiatric illness; (8) neurological disease (e.g., Parkinson’s, multiple sclerosis, stroke with loss of sensory or motor function, or uncontrolled seizures); (9) pregnant; (10) significantly greater pain in a body site other than the knee. Participants provided written informed consent prior to data collection procedures and the study was approved by the institutional review board of both universities.

### Procedures

We have previously reported various components of this large-scale project in recent papers (Cruz-Almeida et al., 2022; Peterson et al., 2022a,b). In short, potential participants underwent an initial phone screening to determine study eligibility for sociodemographic (e.g., age, sex, and race) and health information (e.g., knee pain symptoms). Those eligible were scheduled for a health assessment visit where we obtained informed consent, gathered data on the participant’s health and pain history. Self-reported pain measures (Graded Chronic Pain Scale), a blood draw (epigenetic biomarkers) and a handgrip test were obtained during a second visit that was scheduled approximately 1 week following the initial screening visit.

### Self-reported pain

Graded Chronic Pain Scale (GCPS) was used to assess self-reported pain and provided information on the intensity of the pain experienced (pain intensity subscale, items 1–3), and how much the pain interfered with the participants daily lives (pain interference subscale, items 4–6) during the past 6 months (Von Korff et al., 1992). Calculated scores range from 0 to 100, where higher scores indicate more severe pain intensity and pain interference and were used as a continuous variable in our analysis.

### Handgrip

To determine grip strength, the subject held the dynamometer (Jamar plus digital hand dynamometer; Sammons Preston) in their hand, with the elbow bent at a right angle to the body and the elbow tucked into the side of the body with feet flat on the ground. The handle of the dynamometer was adjusted to fit hand size with the base resting on the first metacarpal, while the handle rested on the middle of the four fingers. The subject then squeezed the dynamometer with maximum isometric effort and maintained the contraction for about 3 s before releasing their grip. No other body movement was allowed. The subject was strongly encouraged to give a maximum effort. Three trials were performed on each hand and the average was recorded.

### Blood collection and processing

The blood sampling and processing method that we perform has been previously reported (Cruz-Almeida et al., 2022; Peterson et al., 2022a,b). In summary, blood samples were collected in a 10 ml K2 EDTA tube that was subsequently used for DNA extraction and methylation analysis. The EDTA tube was centrifuged at 3,000rpm for 10 min and the buffy coat was extracted and transferred to a cryovial for −80-degree storage. To isolate genomic DNA, the frozen buffy coat samples were thawed to 37°C to dissolve homogeneously. ∼200 μl (or 150–200 μl) of sample was lysed in red blood cell (RBC) lysis buffer and centrifuged at 6,000 rpm for 5 min at room temperature. The supernatant was discarded and sodium EDTA solution was added to the pellet and vortex gently to remove any RBC clumps. Homogenate was incubated at 50–55°C with Proteinase K and SDS solution and equal volume of phenol was added, mixed, and centrifuged at 10,000 rpm for 10 min after the incubation period. Supernatant was transferred in a fresh tube and equal volumes of phenol-chloroform-isoamyl alcohol was added, mixed, and centrifuged at 10,000 rpm for 10 min. The supernatant was then transferred to a fresh tube and equal volume of chloroform-isoamyl alcohol was added followed by centrifugation at 10,000 rpm for a further 10 min. Supernatant was transferred in a fresh tube and 1/10th volume of 3 M sodium acetate along with 2 volumes of absolute alcohol was added. The precipitated DNA was washed with 70% ethanol by centrifugation at 10,000 rpm for 5 min. The pellet was air dried and dissolved in Tris-EDTA buffer. The dissolved DNA was qubit quantified and visualized on agarose gel for quality assessment. Sodium Bisulfite conversion and Illumina EPIC methylation array (manufacturer: Illumina, Inc.) was performed.

### DNA methylation age calculation

Similar to our prior work (Cruz-Almeida et al., 2022; Peterson et al., 2022a,b), the raw data generated by Infinium Methylation EPIC array (.idat files) were processed using R package minfi (Aryee et al., 2014). Methylation beta values (percentage of methylation for each CpG site) were obtained and uploaded to the DNA Methylation Age online calculator^1^ (Horvath, 2013) where we followed tutorial recommendations. The data was normalized and the advanced analysis options were used to obtain the predicted epigenetic ages of our participants. To assess biological age difference, we subtracting participant’s chronological age from their epigenetic age to get an age difference, whereby a more positive difference indicates increased epigenetic age acceleration (DNAmGrimAge-Diff). DNAmGrimAge was chosen because it uses an age model built on eight different DNAm-based measures consisting of 1,030 unique CpGs for smoking pack-years as well as a selection of plasma proteins and it is the epigenetic clock most significantly associated with the multidimensional pain experience in persons with knee pain (Cruz-Almeida et al., 2022).

### Statistics

The following analyzes were ran in SPSS v27.0 (IBM Corp, Armonk, NY, USA) and data were checked for distributional form and outliers using z-scores and the standard cut off ± 3SD, anyone outside that range was excluded. Prior to running analyses, the data were cleaned so only those with complete data for the variables of interest (demographics, pain, handgrip and epigenetic profile) were used in the analyses. Independent *t*-tests were used to assess sex differences among variables of interest. Relative strength was calculated by dividing absolute strength (kg) by height (cm) as suggested by Maranhao Neto et al. (2017). The age-adjusted DNAmGrimAge-Diff variable was calculated as the difference between chronological age and DNAmGrimAge. Next, linear regression-based moderated-mediation analyses was employed to observe any effects of sex on the model, with DNAmGrimAge-Diff as the independent variable (X), sex as the moderator (W), GCPS score (i.e., pain intensity and pain intensity) as the mediator (M), and relative hand grip strength (left and right hand averaged) as the dependent variable (Y), with age, race and site as the covariates. a pathway represents is the direct effect of the X variable on the mediator, b pathway is the direct effect of M on Y and the c pathway is the direct effect of X on Y. To overcome potential unmet assumptions commonly found in pathway analysis, bootstrapping procedures were employed for all analyses with 5,000 samples and reported as estimates and standard errors or as 95% bootstrapped confidence intervals.

## Results

### Participants and sex differences

Of the 245 individuals who participated in the parent study, 148 individuals had some degree of pain as reported on the GCPS, complete epigenetic, handgrip, and all covariate data (97 of the original sample did not have complete data). Participant demographics have been previously reported (Peterson et al., 2022a,b; Strath et al., 2022b). Participants in the sample were mostly female (62.2%), evenly distributed non-Hispanic black (46%) and non-Hispanic White (54%) and had a mean age of 57.84 ± 7.72 years. The mean handgrip strength was 10.62 ± 3.92 kg (right hand) and 9.78 ± 3.74 kg (left hand). The mean DNAmGrimAge-Diff was 2.69 ± 5.79 years and the sample had an average pain intensity rating of 14.86 ± 7.57 and pain interference rating of 22.06 ± 35.31. Males and females did not differ in self-reported pain intensity (*p* = 0.176) or pain interference (*p* = 0.113). Differences in DNAmGrimAge-Diff and handgrip strength were found, where males had an older epigenome (DNAmGrimAge-Diff) than females (*p* < 0.001), however, males had greater handgrip strength compared to females in both their absolute (*p* < 0.001) and relative strength (*p* < 0.001) values (see Table 1).

**TABLE 1:** Sex differences among variables of interest.

	Males (56)	Females (92)	*P*-value
GCPS pain intensity	16.11 ± 7.38	14.11 ± 7.63	0.176
GCPS pain interference	28.5 ± 42.38	18.15 ± 29.80	0.113
DNAmGrimAge-Diff (years)	6.34 ± 5.85	0.46 ± 4.49	<0.001
Absolute right hand grip (kgs)	13.77 ± 3.93	8.70 ± 2.38	<0.001
Absolute left hand grip (kgs)	12.92 ± 3.63	7.87 ± 2.19	<0.001
Relative right hand grip (kgs/cm)	0.15 ± 0.05	0.11 ± 0.04	<0.001
Relative left hand grip (kgs/cm)	0.14 ± 0.04	0.10 ± 0.03	<0.001

GCPS, Global Chronic Pain Scale; kgs, kilograms; kgs/cm, kilograms by centimeters.

### Moderated mediation

**FIGURE 1:**
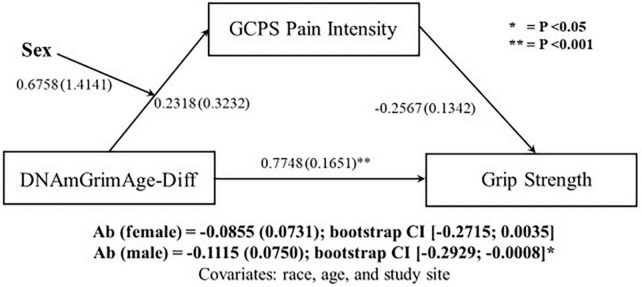
Schematic of moderated-mediation analysis using DNAmGrimAge-Diff as the predictor for grip strength, and Graded Chronic Pain Scale (GCPS) pain intensity as the mediator and sex as the moderator. Ab indicates the indirect pathway (total mediation). Individual regressions along the direct and indirect pathway are reported as beta (SE).

**FIGURE 2:**
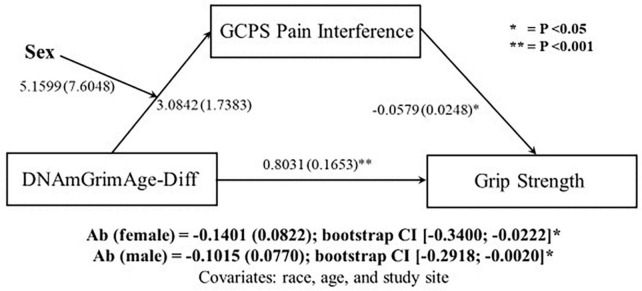
Schematic of moderated-mediation analysis using DNAmGrimAge-Diff as the predictor for grip strength, and Graded Chronic Pain Scale (GCPS) pain interference as the mediator and sex as the moderator. Ab indicates the indirect pathway (total mediation). Individual regressions along the direct and indirect pathway are reported as beta (SE).

Since sex differences were observed in both DNAmGrimAge-Diff and relative handgrip strength, moderated-mediated analyses were performed, with sex as a moderator. The first moderated-mediation exploring the role of pain intensity as a mediator (Figure 1), revealed a significant direct effect of DNAmGrimAge-Diff on relative handgrip strength (β = 0.7748; *p* < 0.001). Furthermore, there was a significant indirect effect of DNAmGrimAge-Diff on relative handgrip strength through GCPS pain intensity in males [β = −0.1115; CI (−0.2929, −0.0008)], but not females [β = −0.0855; CI (−0.2715, −0.0035)]. The second moderated-mediation exploring the role of pain interference as a mediator (Figure 2) revealed a significant direct effect of DNAmGrimAge-Diff on relative handgrip strength (β = −0.8031; *p* < 0.001). Furthermore, there was a significant indirect effect of DNAmGrimAge-Diff on relative handgrip strength through GCPS pain interference in males [β = −0.1015; CI (−0.2918, −0.0020)], and females [β = −0.1401; CI (−0.3400, −0.0222)].

## Discussion

An age-adjusted version of DNAmGrimAge that examines epigenetic age differences (DNAmGrimAge-Diff) has been previously associated with a host of pain-related conditions, lifestyle factors, and clinical biomarkers (Lu et al., 2019; Föhr et al., 2021; Kim et al., 2021; Peterson et al., 2022a; Strath et al., 2022a,b). In other physical function tests, increased epigenetic age acceleration is usually associated with declines in physical function (Lu et al., 2019; McCrory et al., 2020; Föhr et al., 2021; Peterson et al., 2022a); and our moderated-mediation with sex as the moderator and pain intensity as the mediator, revealed a negative association between epigenetic age acceleration and HGS among males only. When we used pain interference as the mediator in the moderated-mediation analysis, we found HGS was negatively associated with epigenetic aging in males and females independently.

A key medical research goal is to identify reliable predictors of mortality by identifying proxy measures of pathological mechanisms that are related to declines in health. Skeletal muscle tissue health is of great interest since skeletal muscle mass is lost at a rate of 0.5–1% per year after age 50 (Mitchell et al., 2012). This clinically significant loss of muscle quality and quantity is associated with a host of age-related complications including frailty, as well as increased morbidity and mortality. Evidence shows that HGS is robustly associated with mortality (Leong et al., 2015), and is thought to be a global proxy of the musculoskeletal system (Lauretani et al., 2003; Snih et al., 2004). As our moderated-mediation results suggest, biological age-related declines in strength occurred in males and females suggesting that HGS could be used as a proxy measure of accelerated aging, providing that we acknowledge sex as a biological variable and consider this in methodological designs. Previous research examining strength performance as a standalone variable, however, has either provided no association with epigenetic aging (McCrory et al., 2020), or demonstrated an association in females only (Peterson et al., 2021). Previously, a stronger association between HGS and impaired activities of daily living has been observed in men than women (Oksuzyan et al., 2010). The aggregate of findings demonstrates a systematic trend in the relationship between age and strength, with strength development peaking in the 4th decade of life and then progressively waning throughout older age with up to 50% of muscle mass being lost by the 8th decade of life even in otherwise healthy adults (Kallman et al., 1990; Trombetti et al., 2016). Further research examining epigenetic aging and musculoskeletal strength should examine an older-aged sample since we used middle-older aged adults within our study sample. Additionally, since males have larger peak muscle mass and greater peak strength than females, it is plausible that this epigenetic aging calculation paired with HGS could be suggestive of a more rapid loss of strength as a product of aging among males reporting higher pain intensity. Alternatively, accelerated biological aging may be due to accelerated musculoskeletal system weakening since males have “more to lose” and those who have chronic pain may undergo accelerated losses of skeletal muscle due to increased risk of sedentary behavior often seen in those with chronic pain. Since we did not measure sedentary behavior caused by chronic pain, further research examining this bidirectional phenomenon needs further review. Additionally, reduced grip strength in our sample could be attributed to pain itself due to fear of movement-evoked pain, slowed recruitment/de-recruitment of motor units, and reduced somatosensory feedback.

Skeletal muscle and its ability to produce force is remarkably plastic, which makes it a highly responsive target tissue for lifestyle changes. For example, changes in DNA methylation that occur with a healthy diet and exercise may be mechanistically involved in slowing down the aging process (Horvath, 2013; Moore et al., 2013; Brown, 2015; Lu et al., 2019). We have previously suggested that chronic pain could be a symptom of the aging body due to its associations with biological aging (Peterson et al., 2022b). Pain is a complex phenomenon that involves many physiological systems and is a major contributor to disability that could affect lifestyle and therefore indirectly hasten biological aging. An increase in sedentary behaviors contributing to a lack of physical activity due to pain related disability can reduce global strength and further accelerate biological aging. Physical activity changes and biological aging in those with chronic pain warrants further investigation.

We acknowledge that there were some limitations to our study, the first being the cross-sectional design. While the current study provides valuable information of the associations between pain, handgrip strength, and epigenetic aging at a given point in time, the study does not effectively assess the relationship over time, nor does it provide any evidence of causality. As such, a strength intervention designed to improve global strength whilst tracking epigenetic modifications may provide further clarification of this relationship. Longitudinal studies should also examine how chronic pain may accelerate aging of the epigenome as well as other biological systems (i.e., telomere length, brain aging etc.) of an individual with chronic pain, especially high impact pain that limits physical performance. Despite reporting chronic pain, our sample was relatively young and healthy compared to prior aging studies. Specifically, our participants were significantly younger (∼58 years old) than those studied previously (∼68–70 years old), with higher levels of physical and cognitive function, and lower levels of disability (Marioni et al., 2015; Föhr et al., 2021). Future studies across the lifespan using a larger sample are needed to examine the consistency of these findings. While the sample was originally recruited on the basis that they had knee osteoarthritis or were at risk from developing knee osteoarthritis due to reported knee pain, the GCPS asked questions on pain in general and were not specific to the knee. As the study population was specific to persons with chronic knee pain, generalizability to other chronic pain conditions is limited. Future studies, including participants with other specific chronic pain conditions are needed to further elucidate the association between pain, biological aging, and strength. Furthermore, using a strength test specific to the knee rather than a global strength test (HGS) may have led to differing results.

Despite these limitations, we were able to provide initial evidence that chronic knee pain, as determined by pain intensity and pain interference, may accelerate epigenetic aging processes that then may ultimately influence global strength in middle to older age adults. Many lifestyle factors, such as diet and exercise both of which can alter our epigenome, are known to have a positive prophylactic effect on muscle morphology and strength (Sharples et al., 2016; Seaborne et al., 2018; Widmann et al., 2019). Interventions that focus on these factors have also shown to be successful in attenuating, and even reversing, the effects of muscle atrophy and improving quality of life and potentially increasing the lifespan (Govindaraju et al., 2018; Campbell et al., 2021). Epigenetic aging may be a useful marker of general health across the lifespan, and may identify those at greatest risk of age-related functional deterioration and death. However, these methods are expensive and a simple handgrip assessment may be a less costly alternative. Continued investigations examining longitudinal impacts on biological aging processes and interactions with physical function in those who have chronic pain may provide causative information for understanding the mechanisms behind pain related disability. In the future, we intend to evaluate how environmental factors, such as exercise and diet, could influence aging via biological aging, particularly within our epigenome.

## Data availability statement

The data analyzed in this study is subject to the following licenses/restrictions: This manuscript utilizes proprietary data. Requests to access these datasets should be directed to YC-A, cryeni@ufl.edu.

## Ethics statement

The studies involving human participants were reviewed and approved by University of Florida Institutional Review Board. The patients/participants provided their written informed consent to participate in this study.

## Author contributions

JP wrote the manuscript. JP and JC conceptualized the research question (introduction), computed the statistical analysis (results), and interpreted the data and critically discussed findings (discussion). AJ, TF, and RF edited and reviewed the manuscript. AR was actively involved in assessing the epigenetic data and reviewed the manuscript. LM and ZH helped with calculations and reviewed the manuscript. YC-A guided the manuscript progress, edited, and reviewed the manuscript. All authors contributed to the article and approved the submitted version.

## Footnotes

1
https://dnamage.genetics.ucla.edu/home
